# Thyroid dysfunction alters gut microbiota composition and alveolar bone levels in female mice

**DOI:** 10.14814/phy2.70817

**Published:** 2026-03-19

**Authors:** Lucia Thompson, Brailey Coulter, Cinnamon VanPutte

**Affiliations:** ^1^ Biological Sciences Southern Illinois University Edwardsville Edwardsville Illinois USA; ^2^ Biomedical and Craniofacial Sciences Southern Illinois University School of Dental Medicine Alton Illinois USA; ^3^ Present address: Molecular and Integrative Physiology University of Illinois Urbana‐Champaign Urbana Illinois USA

**Keywords:** alveolar bone, gut microbiota, gut‐thyroid‐bone axis, periodontal disease, thyroid hormone

## Abstract

Thyroid hormone dysfunction has been implicated in both bone metabolism and gut microbial composition, yet the interplay between thyroid status, gut microbiota, and alveolar bone homeostasis remains unclear. In this study, female C57BL/6J mice were treated with triiodothyronine (T3) and thyroxine (T4) to induce hyperthyroidism or with methimazole (MMI) to induce hypothyroidism. Hyperthyroid mice exhibited decreased alveolar bone levels, measured as greater root exposure between the cementoenamel junction (CEJ) and alveolar bone crest (ABC) (buccal: 41% increase, *p* = 1.566e‐07, 1‐β = 1.0; lingual: 8% increase, *p* = 0.011, 1‐β = 0.71) relative to controls, whereas hypothyroid mice exhibited reduced root exposure (buccal: 23% decrease, *p* = 0.020, 1‐β = 0.65; lingual: 11% decrease, *p* = 0.011, 1‐β = 0.74) relative to controls. 16S rDNA sequencing revealed that both hyper‐ and hypothyroid treatments reduced gut microbial diversity and altered community composition. These findings indicate that thyroid dysfunction can influence both gut microbiota and alveolar bone homeostasis, suggesting a potential gut–thyroid–bone axis. Further investigation is needed to elucidate the mechanistic links between endocrine status, microbial metabolites, and bone remodeling.

## 1 INTRODUCTION

Periodontal disease is a complex, multi‐species microbial condition that can cause chronic inflammation of the periodontium—the tooth‐supporting tissues—leading to alveolar bone loss and eventual tooth loss (Medara et al., 2021), (Pihlstrom et al., 2005). More than one billion individuals are affected worldwide, making it a leading cause of tooth loss (Botelho et al., 2022), (Kassebaum et al., 2017). However, the precise etiology of periodontal disease is still not well‐understood; although dysbiosis of the oral microbial community is a key contributing factor, with elevated salivary levels of keystone periodontopathogens, such as *Porphyromonas gingivalis*, *Prevotella intermedia*, and *Aggregatibacter actinomycetemcomitans*, in patients with periodontal disease (Saygun et al., 2011), (von Troil‐Lindén et al., 1995).

Periodontal disease has been linked to systemic conditions, particularly those associated with gut microbial dysbiosis. Patients with periodontal disease exhibit gut microbial communities that differ from those of healthy individuals (Olsen & Yamazaki, 2019), (Petrukhina et al., 2017). Experimental studies performed exclusively with male mice show that oral colonization with *P*. *gingivalis* can induce gut dysbiosis, alter intestinal barrier integrity, and increase circulating inflammatory markers (Arimatsu et al., 2014), (Giri et al., 2022), (Meilian et al., 2016), (Nakajima et al., 2015). These findings suggest a bidirectional oral‐gut microbiome axis where oral microbes can influence gut microbial composition and systemic physiology (Olsen & Yamazaki, 2019), (Deandra et al., 2023), (Iwauchi et al., 2019), (Virili et al., 2024).

One area of periodontal research that has received increasing attention is the relationship between thyroid dysfunction and periodontal disease (Ni et al., 2025). Thyroid hormones are critical for multiple aspects of intestinal structure and function; they drive intestinal epithelial development, differentiation, and maintenance of a robust barrier in mature intestines. For example, thyroid hormones upregulate intestinal alkaline phosphatase, which degrades bacterial endotoxin (LPS) and protects the barrier from degradation (Malo et al., 2004), (Koyama et al., 2002). Studies have demonstrated that thyroid dysfunction is associated with gut dysbiosis (Christovich & Luo, 2022), (Frau et al., 2017), (Giolito & Plateroti, 2022) and that the gut microbial community of hypo‐ and hyperthyroid patients differs compared to that of euthyroid patients (Ishaq et al., 2017), (Liu et al., 2022), (Su et al., 2020), (Yang et al., 2022), (Zhou et al., 2014).

Thyroid hormones also regulate bone turnover, likely through osteoclasts, which express thyroid hormone receptors and transporters (Krieger et al., 1988), (Lademann et al., 2022), (Lademann et al., 2020), (Usui et al., 2021). In humans, subclinical hyperthyroidism is associated with greater bone loss and increased fracture risk (Segna et al., 2018), (Biondi & Cooper, 2018), (Taylor et al., 2018). Osteoclast‐mediated bone resorption contributes to tooth loss in periodontal disease, and thyroid dysfunction can enhance inflammatory responses and collagen degradation, potentially exacerbating periodontal disease severity (Mazurek‐Mochol et al., 2024), (Feitosa et al., 2009), (Shcherba, Miz, et al., 2021), (Shcherba et al., 2020). Clinical observations further suggest that thyroid dysfunction may be linked to increased gingival bleeding, gum recession, and alveolar bone loss (Chrysanthakopoulos & Chrysanthakopoulos, 2016), (Monea et al., 2015), (Molloy et al., 2004).

Induced hypo‐ or hyperthyroidism combined with periodontal disease in male rat models amplifies pro‐inflammatory mediators in the periodontium compared to rats with periodontal disease alone (Shcherba, Krynytska, et al., 2021). Consequently, male rats treated to induce periodontitis with hyper‐ or hypothyroidism also exhibit disrupted morphology of the periodontium (Shcherba et al., 2022), (Shcherba et al., n.d.). Though periodontal disease is more common in males, (Shiau & Reynolds, 2010) thyroid disorders are more highly prevalent in females (Vanderpump, 2019). Studies utilizing female rodent models of thyroid dysfunction with periodontal disease are severely lacking. Thus, this study aimed to address this knowledge gap by using female mice. Besides rats, mice have also been used in numerous studies as a valid model for studying periodontal disease (Arce et al., 2023), (Graves et al., 2008), (Abe & Hajishengallis, 2013), (Haacke et al., 2025).

Despite these observations, the relationship between thyroid function, gut microbial composition, and baseline alveolar bone homeostasis remains poorly understood. In this study, we induced hyperthyroidism or hypothyroidism in female C57BL/6J mice to investigate how thyroid dysfunction affects gut microbiota and alveolar bone. We hypothesized that hyperthyroidism would decrease alveolar bone levels, measured as greater root exposure between the cementoenamel junction (CEJ) and alveolar bone crest (ABC), whereas hypothyroidism would reduce root exposure. We further hypothesized that both hyper‐ and hypothyroid states would alter gut microbial diversity and community composition.

## 2 METHODS

Animal care procedures and experiments designed for this study were approved by the Institutional Animal Care and Use Committee of Southern Illinois University Edwardsville (IACUC protocols #1167/2570). Thirty‐eight 3‐week‐old female C57BL/6J mice were purchased from The Jackson Laboratory (Bar Harbor, ME, USA; RRID:IMSR_JAX:000664) and maintained at 25°C with a 14 L:10D photoperiod. Five mice were housed in each SuperRat 1400 AllerZone™ Micro‐Isolator® unit with irradiated SSP ALPHA DRI‐IRRAD paper bedding. Mice were fed irradiated 53WU PICOLAB Rodent 20 feed and water ad libitum.

Following a 2‐week acclimation period, mice were treated with thyroid hormones for 6 weeks or methimazole for 10 weeks. As the experimental protocols and quantitative thyroid hormone measurements have been reported previously, (Thompson et al., 2024) these data are not reproduced in the present manuscript. Methimazole was administered for a longer timeframe to ensure consistent suppression of endogenous thyroid hormone production and sufficient depletion of circulating and tissue stores. Thyroid hormone treatment was limited to a shorter interval. This approach was chosen because thyroid hormone produces physiological effects quickly, and prolonged supraphysiological exposure can lead to unnecessary toxicity. Our aim was to assess acute responses rather than model chronic thyrotoxicosis. Hormonal status was verified by measuring plasma T4, and animals were monitored daily for any signs of adverse effects.

To induce hyperthyroidism, drinking water contained 2 μg/mL of T4 (Catalog ICN15214501; Fisher Scientific) and 0.5 μg/mL of T3 (Catalog ICN19458580; Fisher Scientific), stabilized with 0.1% w/v bovine serum albumin (BSA) (Catalog BP1600‐100; Fisher Scientific) and 4 mM of NaOH (Catalog S318‐500; Fisher Scientific), and flavored with 0.2% w/v grape Kool‐Aid and 0.4% w/v sucralose (Splenda®) (Feitosa et al., 2009), (Thompson et al., 2024). Ten mice received this treatment; five control mice received water containing only BSA, NaOH Kool‐Aid, and sucralose. To induce hypothyroidism, drinking water contained 0.1% w/v methimazole (MMI) (Catalog 11–101‐1115; Fisher Scientific) with the same flavoring (Thompson et al., 2024), (Hoefig et al., 2015), (Zhou et al., 2015); 10 mice received this treatment with five control mice receiving flavored water without MMI. Because MMI‐induced hypothyroidism and TH treatment‐induced hyperthyroidism produce greater physiological variability and carry an expected risk of treatment‐related attrition, additional animals were assigned to the experimental groups to ensure adequate statistical power for detecting biologically relevant effects. Control animals were limited to the minimum necessary to establish baseline measures, with appropriate use of historical control data.

As previously described (Thompson et al., 2024), fecal samples were collected following isolation and stored at −80°C. Mice were anesthetized via isoflurane exposure using the open‐drop method. Whole blood was collected by cardiac puncture, plasma was separated and stored at −80°C. Mice were euthanized by cervical dislocation, and hemimandibles were dissected, cleaned, and stored at 4°C.

To confirm induction of hyper‐ and hypothyroid states, circulating total T4 was quantified from collected plasma samples. These concentrations were measured using either a competitive T4 ELISA kit (MBS700042; MyBioSource Inc., San Diego, CA, USA) or a Rat Thyroid Magnetic Bead Panel (RTHYMAG‐30 K; MilliporeSigma). Values were interpolated using either a 4PL regression analysis available through “Four Parameter Logistic Curve” online data analysis tool, MyAssays Ltd., http://www.myassays.com/four‐parameter‐logistic‐curve.assay, or a cubic spline curve using xPONENT® Software Solutions for Luminex® Systems. Data were assessed with the Shapiro–Wilk test for normality, an *F*‐test for variance, and differences were assessed using Student's *t*‐test.

Hemimandibles were treated with 7.5% NaClO solution for 1–3 h to remove remaining tissue, rinsed, and dried. Dry hemimandibles were stained with 1% methylene blue for 5 min, rinsed, and dried. Each stained hemimandible was placed on a bed of sesame seeds in a 30 × 15 mm Petri dish to orient the tissue level with the base of the microscope. Images of both buccal and lingual sides of each hemimandible were collected using a LaxCo dissection scope fitted with a camera at 20× magnification.

In diagnostic periodontal research, disease severity is commonly assessed using several criteria, including horizontal bone loss (Abe & Hajishengallis, 2013), (Zhou et al., 2023), (Catunda et al., 2021), (Marchesan et al., 2018), (Baker et al., 2002). Horizontal bone loss—referred to in this study as alveolar bone levels—is defined as bone resorption that occurs parallel to a line connecting the cementoenamel junctions of adjacent teeth, resulting in a uniformly reduced height of the alveolar bone crest. Alveolar bone levels were assessed by using the ImageJ software (Schneider et al., 2012) to quantify total area of root exposed between the CEJ and ABC of first and second molars. Length of the first and second molars was also recorded. Measurements were normalized to molar length and converted to millimeters (524.1374 pixels/mm). Although micro‐CT provides high‐resolution imaging of bone morphology, its use was not feasible within the constraints of our current funding. We therefore employed established alternative methods (Meilian et al., 2016), (Davis et al., 2022), (Settem et al., 2021), (Azambuja et al., 2012), (Liberman et al., 2011) that offer sufficient sensitivity for the stated hypotheses of this study. Data normality was determined by the Shapiro–Wilk test, variance was determined with the *F*‐test and differences were assessed using Student's *t*‐test except for lingual measurements from TH‐treated mice in which a Mann–Whitney *U* test was utilized since data were not normally distributed.

Using a QIAamp PowerFecal Pro DNA kit (Catalog 51,804; QIAGEN), microbial DNA was isolated from a single fecal pellet per mouse. Although oral microbiota is increasingly recognized as a contributor to periodontal and systemic physiology, we did not evaluate oral microbial composition in this work due to financial limitations. Moving forward, both gut and oral microbiota will be evaluated. For 16S rDNA microbiome analysis, fecal pellets were collected from control animals before exposure to Kool‐Aid and sucralose as a control. Throughout the 2 weeks preceding collection, these animals consumed only plain drinking water. DNA quality (260/280 ratio 1.8–2.0) was verified, and samples were sent to Novogene Corporation for 16S rDNA sequencing. V4–V5 hypervariable regions were amplified using primers 5′‐GTGCCAGCMGCCGCGGTAA‐3′ and 5′‐CCGTCAATTCCTTTGAGTTT‐3′. Sequencing was paired‐end Illumina, OTUs were clustered at 97% similarity using UPARSE (Edgar, 2013), and annotated with Silva 138.1 (Quast et al., 2013). Alpha diversity indices (Chao1, Shannon, Simpson) and beta diversity (Weighted UniFrac, Bray–Curtis dissimilarity) were calculated using QIIME v1.9.1 (Caporaso et al., 2010). Differences between groups were assessed using a one‐way ANOVA with Tukey post‐hoc HSD. Historical data is represented by weighted UniFrac, where the phylogenetic distance between two communities is calculated by utilizing the relative abundance of microbes in each group; differences were determined utilizing the Mann–Whitney *U* test since the data were not normally distributed (Lozupone et al., 2011).

Statistical analyses were performed using R v4.3.1 (R Core Team, 2023). Cohen's d was calculated to determine the effect size of two group comparisons, and Cohen's f was calculated to determine the effect size of three group comparisons. Then, power analyses of t‐tests and ANOVA tests were performed. The statistical power (1‐ꞵ) for each analysis was reported along with *p*‐value.

## 3 RESULTS

### 3.1 Study design and validation

To verify that thyroid hormone supplementation and administration of methimazole effectively induced thyroid dysfunction, circulating total T4 levels were assessed using plasma samples. Addition of T4 and T3 to drinking water significantly (*p* = 1.943e‐06, 1‐β = 1.0) increased circulating levels of total T3 and T4 (Thompson et al., 2024). This observation was consistent with a potential hypermetabolic state characteristic of hyperthyroidism, as experimental group cages required changing at 2‐day intervals compared to the standard 7‐day schedule for control animals. Addition of 0.1% w/v of MMI to drinking water significantly (*p* = 8.391e‐05, 1‐β = 1.0) reduced circulating levels of total T4 (Thompson et al., 2024). These data were previously reported in Thompson et al. (2024). It is also important to note that addition of BSA and NaOH to drinking water to improve thyroid hormones solubility did not influence thyroid function, as circulating total T4 levels measured from both sets of control mice were not significantly different (*p* = 0.8948, 1‐β = 0.05).

### 3.2 Alveolar bone findings

**FIGURE 1:**
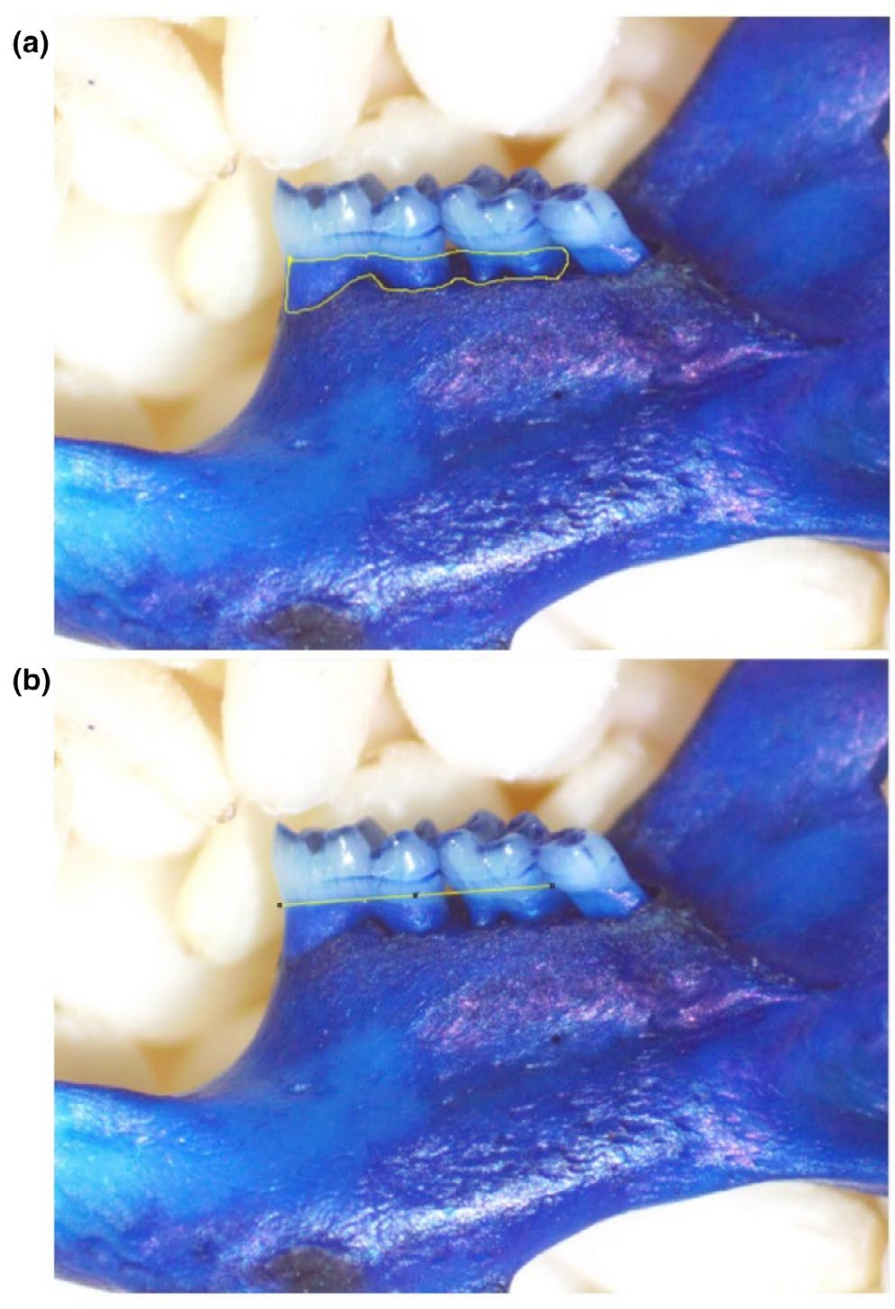
Measurement of Root Exposure. (a) In ImageJ, the area between the CEJ and the ABC of the first two molars was measured. (b) The length between the CEJ of the first two molars was measured.

**FIGURE 2:**
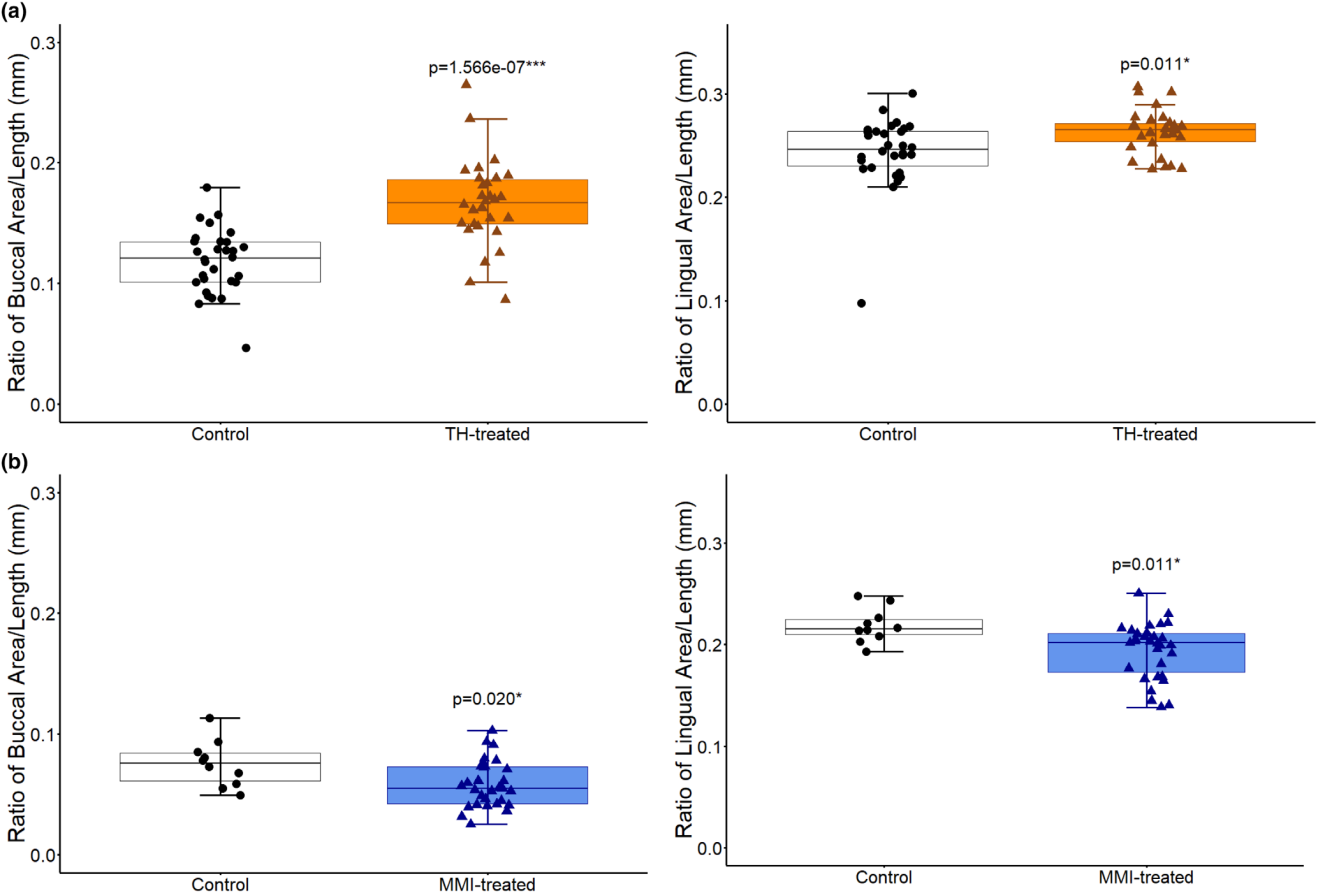
Area of Root Exposure Normalized to Molar Length. (a) TH‐treated mice exhibited greater root exposure relative to controls (Control *n* = 30, TH‐treated *n* = 30; Buccal: 41% increase, *p* = 1.566e‐07, 1‐β = 1.0; Lingual: 8% increase, *p* = 0.011, 1‐β = 0.71) (b) MMI‐treated animals showed reduced root exposure (Control *n* = 10, MMI‐treated *n* = 30; Buccal: 23% decrease, *p* = 0.020, 1‐β = 0.65; Lingual: 11% decrease, *p* = 0.011, 1‐β = 0.74). Each point represents the individual measurements of each hemimandible. Boxes show the IQR from Q1 to Q3, horizontal lines indicate the median, and whiskers extend to Q1 − 1.5× IQR and Q3 + 1.5× IQR. All statistical comparisons were performed using Student's *t*‐test, except for lingual measurements of TH‐treated mice, which used a Mann–Whitney *U* test (*p < 0.05, ***p < 0.001).

**FIGURE 3:**
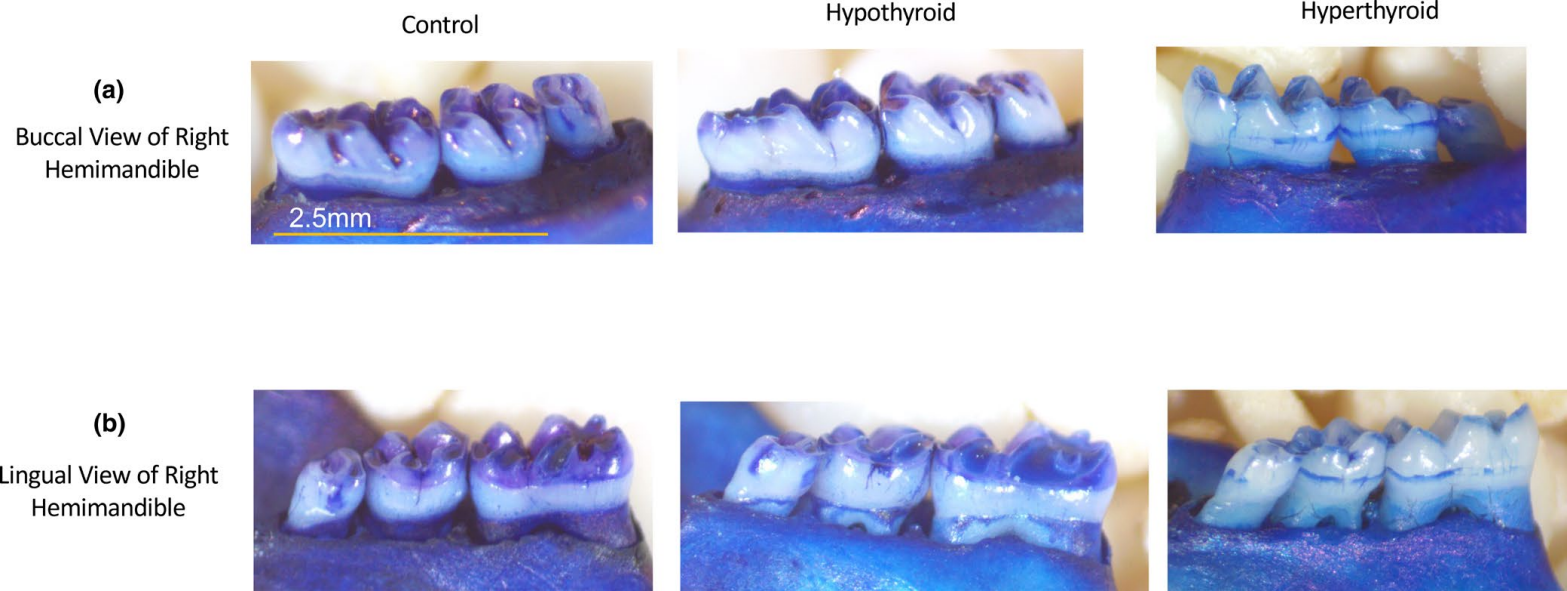
Representative Photographic Images of Root Exposure. (a) Buccal surface images of right hemimandible illustrate reduced root exposure in MMI‐treated mice and increased exposure in TH‐treated mice. The average length of the first two molars was 2.5 mm, represented by a scale bar. (b) Lingual surface images show a similar pattern to the buccal surface.

To elucidate the relationship between thyroid function and alveolar bone levels, area of root exposure between the CEJ and ABC was quantified in mice treated with either thyroid hormone supplementation or methimazole administration (Figure 1). Decreased alveolar bone levels were observed in TH‐treated mice compared to controls, shown by increased area of root exposure relative to tooth size on both buccal and lingual sides (buccal: 41% increase, *p* = 1.566e‐07, 1‐β = 1.0; lingual: 8% increase, *p* = 0.011, 1‐β = 0.71) (Figures 2a and 3). Conversely, MMI‐treated mice exhibited reduced area of root exposure compared to controls on both buccal and lingual sides (buccal: 23% decrease, *p* = 0.020, 1‐β = 0.65; lingual: 11% decrease, *p* = 0.011, 1‐β = 0.74) (Figures 2b and 3).

### 3.3 Microbiota findings

**FIGURE 4:**
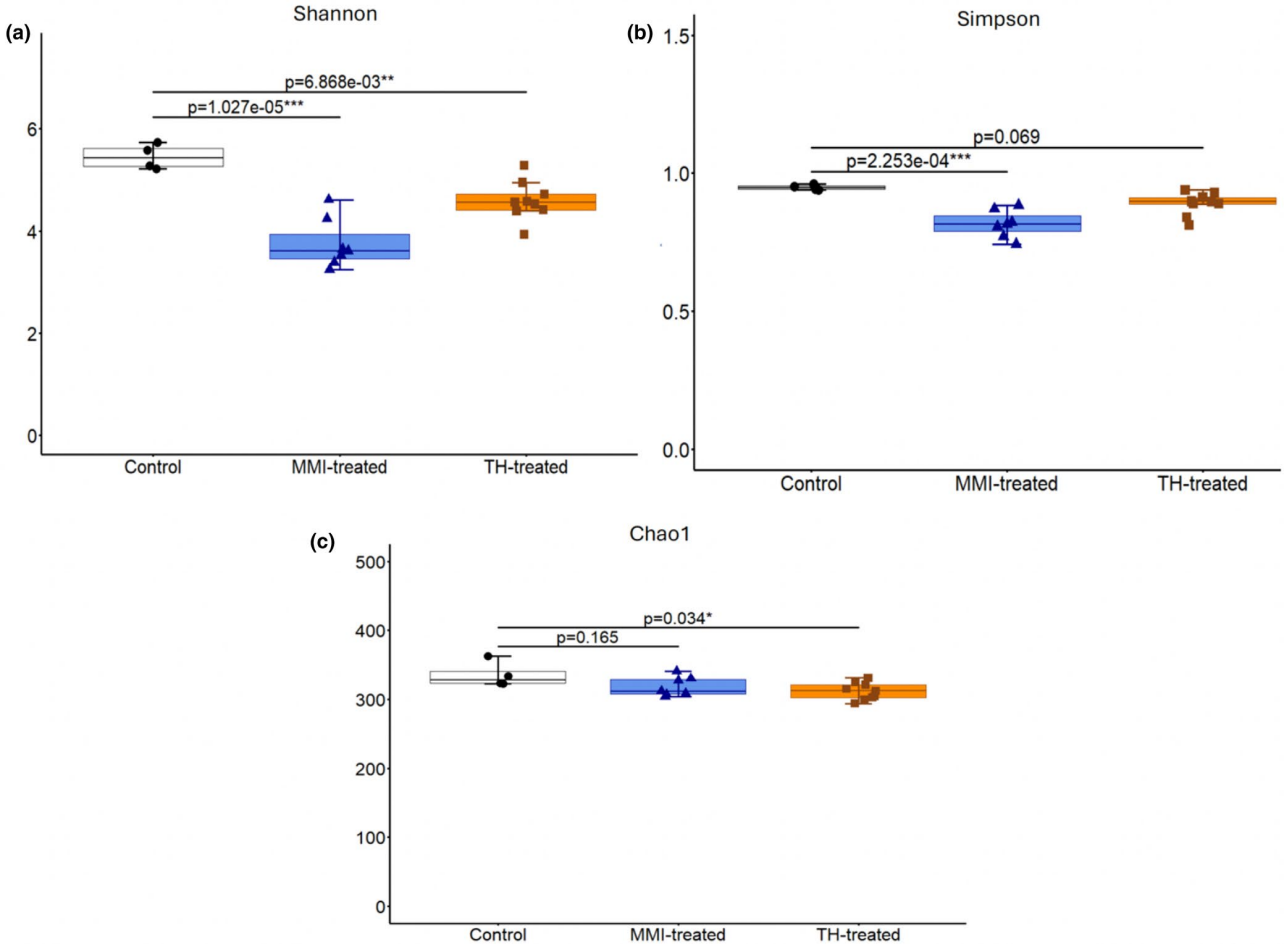
Thyroid Dysfunction and Gut Microbial Alpha Diversity. (a) Alpha diversity was lower in both TH‐treated (*n* = 9) and MMI‐treated (*n* = 7) mice compared with controls (*n* = 4), according to the Shannon index (Control‐TH: *p* = 6.868e‐03; Control‐MMI: *p* = 1.027e‐05; 1‐β = 1.0). (b) Using the Simpson index, only MMI‐treated animals demonstrated decreased alpha diversity (Control‐MMI: *p* = 2.253e‐04; 1‐β = 1.0) (c) Using the Chao1 index, only TH‐treated animals demonstrated decreased diversity (Control‐TH: *p* = 0.034, 1‐β = 0.62). Boxes show the IQR, horizontal lines represent the median, and whiskers extend to Q1–1.5× IQR and Q3 + 1.5× IQR. Differences between groups were assessed using a one‐way ANOVA with Tukey post‐hoc HSD (*p < 0.05, **p < 0.01, ***p < 0.001).

Alpha diversity indices were calculated to assess biodiversity within experimental groups. Shannon and Simpson indices were used to estimate distribution and evenness within bacterial populations, while the Chao1 index was used to estimate abundance. Both TH‐ and MMI‐treatments reduced alpha diversity according to the Shannon index (Control‐TH: *p* = 6.868e‐03; Control‐MMI: *p* = 1.027e‐05; 1‐β = 1.0) relative to control mice (Figure 4a). MMI‐treatment reduced alpha diversity according to the Simpson index (Control‐MMI: *p* = 2.253e‐04; 1‐β = 1.0) (Figure 4b), and TH‐treatment reduced alpha diversity according to the Chao1 index (Control‐TH: *p* = 0.034; 1‐β = 0.62) (Figure 4c).

**FIGURE 5:**
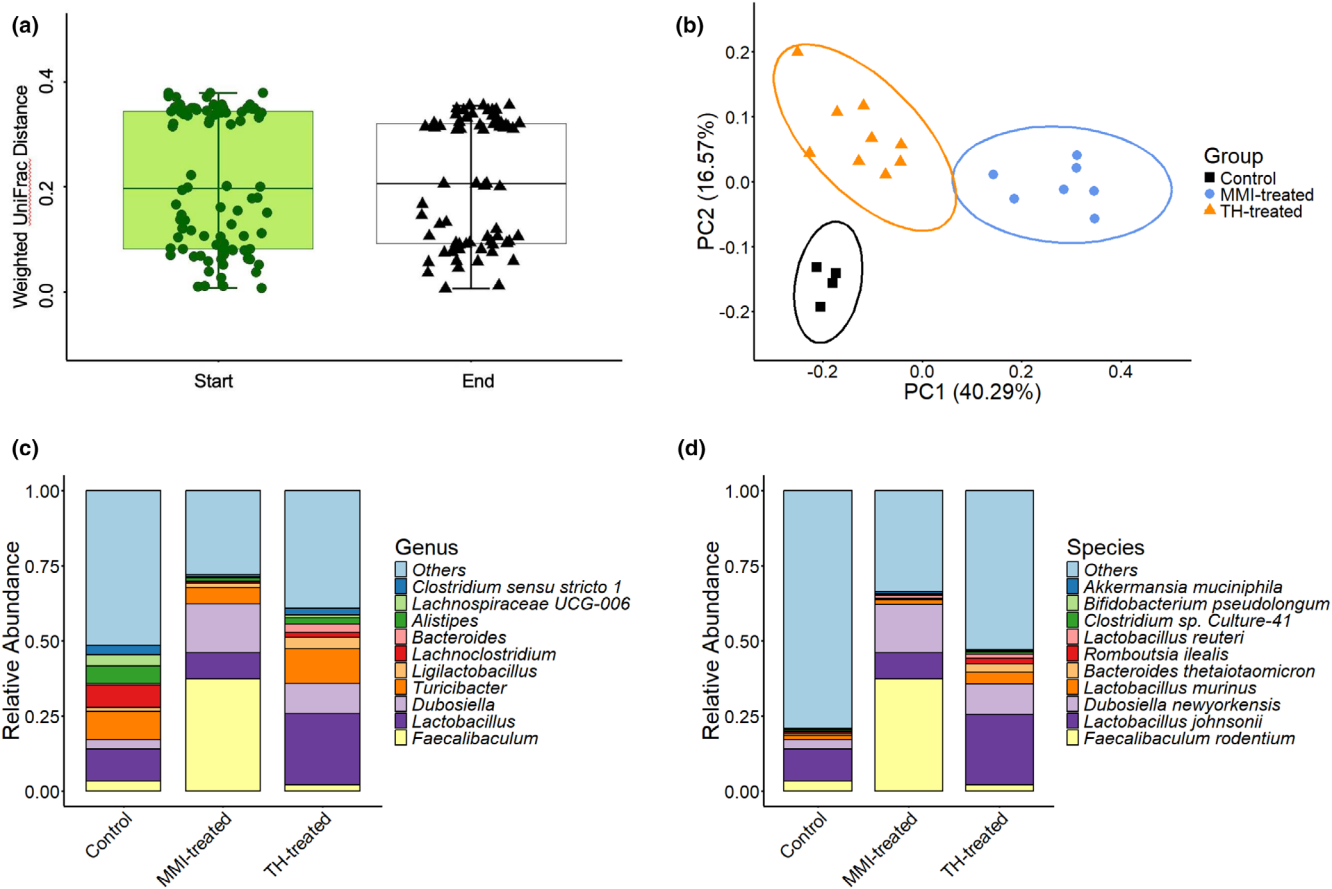
Thyroid Dysfunction and Gut Microbial Beta Diversity and Composition. (a) Weighted UniFrac distances of historical data in our lab illustrate no gut microbiota differences due to consumption of Kool‐Aid and sucralose (Mann–Whitney *U* test; *p* = 0.2465, 1‐β = 0.05). “Start” mice (*n* = 4) received plain drinking water for 2 weeks and “end” mice (*n* = 3) received flavored water for 6 weeks. Each data point is a distance matrix value, which represents how different two microbial communities are based on their abundance and phylogenetic relationship. (b) Bray‐Curtis distances from the present study showed clear compositional differences between experimental and control animals. Each individual data point represents one mouse. If the community composition of the samples is more similar, their distances in the PCoA map are closer. Ellipses represent groupings, showing data clusters and their spread in the reduced dimensions (95% confidence region). Tighter ellipses mean less internal variation within each group (more homogeneity), while little overlap of ellipses suggests differences between TH‐treated and MMI‐treated compared to controls. (c, d) Relative abundances of various genera (c) and species (d) of bacteria were differentially influenced by experimental treatment. (TH‐treated *n* = 9, MMI‐treated *n* = 7, control *n* = 4).

Beta diversity indices were calculated to quantify the relative diversity between microbial communities. Control data represents baseline results from animals receiving only plain drinking water. However, historical data from our lab suggests that gut microbiota are not affected by the addition of Kool‐Aid and sucralose based on equivalent weighted UniFrac distances (Figure 5a), but we cannot draw any conclusions about the addition of NaOH and BSA. Bray–Curtis dissimilarity matrices showed distinct clustering between control, MMI‐treated, and TH‐treated mice, plotted via principal coordinates analysis (PCoA) (Figure 5b). MMI‐ and TH‐treated microbial communities exhibited no overlap with controls within the 95% confidence interval, demonstrating unique community composition.

Analysis of relative abundance revealed specific taxa affected by treatment. Both TH‐ and MMI‐treatment increased *Dubosiella*, particularly *D. newyorkensis* (Figure 5c,d). Both treatments decreased *Lachnoclostridium* and *Alistipes* (Figure 5c). Some taxa responded differently depending on treatment: *Lactobacillus* and *Turicibacter* decreased with MMI but increased with TH, whereas *Faecalibaculum* increased with MMI but decreased with TH (Figure 5c).

## 4 DISCUSSION

### 4.1 Altered thyroid status affects alveolar bone

This study provides evidence that thyroid hormone dysfunction modulates baseline alveolar bone homeostasis. TH supplementation effectively induced a hyperthyroid‐like state by increasing circulating total T4 nearly threefold, while MMI treatment effectively induced a hypothyroid‐like state by reducing circulating total T4 nearly 50% (Thompson et al., 2024). Hyperthyroidism is associated with excessive bone resorption (Bassett & Williams, 2016), (Kshirsagar et al., 2018) while hypothyroidism is associated with reduced bone turnover rate (Mosekilde & Melsen, 1978), which can be accelerated by treatment with L‐thyroxine (Meier et al., 2004), (Stall et al., 1990). Areas of root exposure between CEJ and ABC were quantified as a proxy for the inverse of alveolar bone thickness (Rojo‐Sanchis et al., 2019). Increased root exposure in TH‐treated mice (Figure 2a) may reflect thinner facial bone and higher bone turnover, whereas reduced root exposure in MMI‐treated mice (Figure 2b) suggests thicker facial bone and lower turnover. These changes occurred without induced periodontal disease, supporting a role for thyroid hormones in regulating baseline alveolar bone homeostasis.

### 4.2 TH treatment perturbs gut microbiota

16S rDNA sequencing analysis revealed that induced thyroid dysfunction significantly shapes gut microbial diversity (Figure 4) and composition (Figure 5). Although mice are coprophagic, this behavior is consistent across treatment groups, permitting valid analysis. Mice are a rigorous and widely accepted model for studying gut microbial composition via cecal and fecal contents (Wang et al., 2025), (Guo et al., 2022), (Borin et al., 2023). Microbiota analysis revealed decreased species richness and evenness in gut microbial communities of mice treated with TH, BSA, NaOH, Kool‐Aid, and sucralose for 6 weeks (Figure 4). A decrease in microbial diversity is often associated with dysbiosis and has been observed in various systemic inflammatory conditions, including autoimmune thyroid diseases (Zhao et al., 2018). *Faecalibaculum*, which has anti‐inflammatory properties, decreased in TH‐treated mice, whereas *Lactobacillus*, a probiotic, increased (Figure 5c). Thyroid hormones can influence short‐chain fatty acid (SCFA) production, lowering gut pH and favoring *Lactobacillus* growth (Mendoza‐León et al., 2023), (Liu et al., 2021), (Tannock, 2004).

### 4.3 MMI treatment perturbs gut microbiota

Treatment with MMI, Kool‐Aid, and sucralose for 10 weeks also altered gut community structure and evenness (Figures 4 and 5). Relative abundance of the genus *Faecalibaculum* increased, whereas *Lachnoclostridium* decreased (Figure 5c), which is consistent with previous observations in thyroid disorder patients (Monea et al., 2015), (Zhao et al., 2018). Both genera produce SCFAs, suggesting potential links between microbial metabolites and bone turnover.

Given that both thyroid hormones and gut‐derived metabolites influence systemic immune regulation and bone remodeling, disruptions in the gut–thyroid–bone axis could modulate the severity of periodontal bone loss. Along with thyroid dysfunction, rodent models can be treated to induce periodontal disease via suture placement around a molar or by oral inoculation with the periodontopathogen *P. gingivalis* (Lin et al., 2024), (Graves et al., 2008), (Abe & Hajishengallis, 2013), (Zhang et al., 2014). Previous studies inducing periodontitis along with thyroid dysfunction in male rats have observed increased inflammation and perturbed morphology of the periodontium (Feitosa et al., 2009), (Shcherba, Krynytska, et al., 2021), (Shcherba et al., 2022), (Shcherba et al., n.d.). As the subject of a future study, induction of hyper‐ or hypothyroidism along with periodontal disease in female rodent models could allow researchers to determine whether decreased alveolar bone levels attributed to periodontal disease are exacerbated or alleviated.

There are limitations to our study. One limitation is the absence of oral microbiota analysis. This decision reflects both financial constraints and our focus on characterizing endocrine and tissue responses. Future studies incorporating microbial and metabolite profiling will be important for more fully defining potential microbiome‐mediated mechanisms. A second limitation is that micro‐CT imaging was not performed, again reflecting financial constraints. However, the alternative methods used are well established and provide reliable measures for interpreting bone outcomes (Davis et al., 2022), (Settem et al., 2021), (Azambuja et al., 2012), (Liberman et al., 2011) (Meilian et al., 2016), (Rojo‐Sanchis et al., 2019). Future studies will aim to utilize micro‐CT imaging.

Though thyroid disease has a higher prevalence in females (Vanderpump, 2019), periodontal disease appears to have a higher prevalence in male populations (Shiau & Reynolds, 2010). Future studies should include male mice in addition to females to identify possible sex differences in alveolar bone levels and gut microbiota composition. Reproductive hormones play a role in bone turnover, as estrogen inhibits bone resorption (Kameda et al., 1997) and testosterone promotes both bone resorption and formation (Shigehara et al., 2021). Biological sex also influences the composition of gut microbiota, as male and female mice exhibit distinct microbial communities before and after sexual maturity (de la Ortiz‐Alvarez Campa et al., 2024).

## 5 CONCLUSIONS

Both hyper‐ and hypothyroid treatments induced unique gut microbial community compositions in female mice, while hyperthyroidism increased root exposure and hypothyroidism decreased root exposure of the first two molars. It is possible that a bidirectional relationship between gut microbial composition and thyroid status is mediated through immune function and intestinal barrier integrity; however, more research is needed. Dispersal of bacterial metabolites and inflammatory cytokines throughout the circulation can modulate alveolar bone homeostasis. Indeed, there is extensive evidence that host inflammatory markers are responsible for the progression of periodontal disease (Meilian et al., 2016), (Usui et al., 2021), (Dyke et al., 2020), (Pacifici, 2018), (Yucel‐Lindberg & Båge, 2013). Following initiation of periodontal disease, oral periodontopathogens can be swallowed, disrupting the gut microbiota and weakening the intestinal barrier. Because thyroid dysfunction influences bone turnover rate and is associated with gut microbial dysbiosis, in the future, patients' thyroid status may be considered when clinically evaluating periodontal disease progression.

## AUTHOR CONTRIBUTIONS

LT, BC, and CVP all contributed equally to design, data acquisition, analysis, and interpretation, drafting and revising the work. LT, BC, and CVP all approved the final version of this manuscript and qualify for authorship. CVP agrees to be accountable for all aspects of the work in ensuring that questions related to the accuracy or integrity of any part of the work are appropriately investigated and resolved.

## FUNDING INFORMATION

SIUE Graduate school RGGS (LT and BC). SIU SDM Advanced Initiative Award (AIA24‐02; to CVP).

## CONFLICT OF INTEREST STATEMENT

The authors declare no conflicts of interest.

## ETHICS STATEMENT

Animal care procedures and experiments designed for this study were approved by the Institutional Animal Care and Use Committee of Southern Illinois University Edwardsville (IACUC protocols #1167/2570). The experiments described were conducted in compliance with the US National Research Council's ‘Guide for the Care and Use of Laboratory Animals’, the US Public Health Service's ‘Policy on Humane Care and Use of Laboratory Animals’ and ‘Guide for the Care and Use of Laboratory Animals’.

## Data Availability

The data that support the findings of this study are available from the corresponding author upon reasonable request.
